# Mifepristone Increases Life Span in Female *Drosophila* Without Detectable Antibacterial Activity

**DOI:** 10.3389/fragi.2022.924957

**Published:** 2022-07-22

**Authors:** Gary N. Landis, Luke Riggan, Hans S. Bell, William Vu, Tianyi Wang, Ina Wang, Felicia I. Tejawinata, Sebastian Ko, John Tower

**Affiliations:** Molecular and Computational Biology Section, Department of Biological Sciences, University of Southern California, Los Angeles, CA, United States

**Keywords:** aging, steroid, mifepristone, bacteria, microbiome, *Drosophila*

## Abstract

Mifepristone dramatically increases the life span of mated female *Drosophila* while reducing the expression of innate immune response genes. Previous results indicated that mifepristone also reduced the load of aero-tolerant bacteria in mated females. Experiments were conducted to further investigate the possible role of bacteria in mifepristone life span effects. Life span was assayed in flies grown from sterilized eggs on autoclaved media and in normally cultured controls in two independent assays. Sterilization increased mated female life span (+8.3% and +57%, respectively), and the effect of mifepristone was additive (+53% and +93%, respectively). High-throughput sequencing of 16S sequences revealed that sterilization reduced the abundance of multiple species and the classes *Bacteroidia*, *Bacilli*, *Actinobacteria*, and *Cytophagia.* By contrast, mifepristone caused no decreases and instead increased the abundance of three species*.* Five aero-tolerant bacterial species were cultured from extracts of mated female flies, including both Gram-positive and Gram-negative species (*Acetobacter sicerae, Enterococcus faecalis, Lactobacillus plantarum, Serratia rubidea,* and *Paenibacillus glucanolyticus*). There was no detectable effect of mifepristone on the growth of these bacteria *in vitro*, indicating that mifepristone does not have a direct antibiotic effect. To test if antibiotics could mimic the effects of mifepristone *in vivo*, mated female flies were treated throughout adult life span with high concentrations of the individual antibiotics doxycycline, ampicillin, kanamycin, and streptomycin, in replicate experiments. No significant effect on life span was observed for ampicillin, kanamycin, or streptomycin, and an inconsistent benefit was observed for doxycycline. Finally, supplementation of media with *Enterococcus faecalis* did not alter adult female life span in the presence or absence of mifepristone. Taken together, the results indicate the life span benefits of mifepristone are not due to an antibiotic effect.

## Introduction

Mifepristone (RU486) is a synthetic steroid with a broad range of therapeutic applications. Mifepristone antagonizes the progesterone receptor, enabling its use as an abortifacient and for prophylactic birth control (Baulieu, 1997; Chen et al., 2014; Beaman et al., 2020), as well as for the treatment of uterine fibroids (Islam et al., 2020). Mifepristone also antagonizes the glucocorticoid type II receptor, enabling its use in the treatment of Cushing’s disease (hypercortisolism) (Tritos and Biller, 2019; Brown et al., 2020). Mifepristone is under study for the treatment of numerous types of cancers, including glioma (Kapperman et al., 2018; Alvarez et al., 2021; Check and Check, 2021; Llaguno-Munive et al., 2021; Serritella et al., 2022; Zhao et al., 2022). Mifepristone also shows promising results in the treatment of depression (Block et al., 2018). Several studies report antiobesity and antidiabetic effects of mifepristone in humans and mice (Gross et al., 2010; Bernal-Sore et al., 2018; Gubbi et al., 2021). For example, mifepristone improved insulin sensitivity and adiponectin levels in mice fed with a high-fat diet and caused adiponectin release from cultured adipocytes that was dependent upon PPARγ (Hashimoto et al., 2013). Consistent with those observations, mifepristone is a mammalian PPARγ agonist that activates the expression of PPARγ target genes (Lin et al., 2012; Wu et al., 2018).

Mifepristone was first used in *Drosophila* as the trigger for the conditional gene expression system called Gene-Switch (Osterwalder et al., 2001; McGuire et al., 2004; Ford et al., 2007). Gene-Switch is an engineered transcription factor that includes the regulatory domain from the human progesterone receptor. Gene-Switch is activated upon binding to mifepristone, allowing for conditional expression of target genes simply by feeding flies mifepristone (Wang et al., 1994). While using the Gene-Switch system, we observed that mifepristone had significant effects on female life span in control flies that did not contain a target gene, as well as in wild-type strain flies (Landis et al., 2015). Mifepristone dramatically increases the life span of mated females (approximately +50–100%) and also increases life span in virgin females (approximately +15–30%), with the greatest effects observed in strains that have long starting life spans (Landis et al., 2015). Interestingly, mifepristone did not significantly affect life span in males. Mifepristone also significantly increased median life span in mated *C. elegans* hermaphrodites, in two out of three trials (+6.7% and +13%, respectively), and in the combined data of the three trials (+15%), suggesting the mechanisms may be evolutionarily conserved (Landis et al., 2021a).

In female *Drosophila*, mating and male sex peptide (SP) hormone cause increased juvenile hormone (JH) and ecdysone hormone levels. JH and ecdysone induce intestinal stem cell (ISC) proliferation, midgut hypertrophy, and increased AA and lipid metabolism (Ahmed et al., 2020; Zipper et al., 2020; White et al., 2021), which supports increased egg production (Reiff et al., 2015; Sieber and Spradling, 2015). The midgut hypertrophy, increased AA metabolites and lipids, inflammation, and decreased life span caused by mating and SP can each be blocked by feeding the mated females mifepristone (Landis et al., 2015; Tower et al., 2017; Landis et al., 2021a; Landis et al., 2021b). Transcriptomics analysis indicated that mifepristone downregulates genes in the midgut that are negatively correlated with life span and upregulates genes in the nervous system that are positively correlated with life span (Landis et al., 2015; Landis et al., 2021a). Metabolomics assays revealed that in both mated females and virgins, mifepristone reduces the levels of tryptophan metabolites (Landis et al., 2021b). Remarkably, mifepristone uncouples *Drosophila* life span from food intake. Mifepristone increased the life span of virgin females fed JH analog methoprene by +80%, while simultaneously doubling food intake (Landis et al., 2021a). Similarly, mifepristone can increase the life span of virgin females on both normal and high-fat diets while simultaneously increasing food intake (Landis et al., 2021b). Taken together, the data suggest that mifepristone reduces gut metabolism and midgut hypertrophy that would otherwise shorten life span. Because the fly gut harbors a complex bacterial microbiome, and this microbiome has been implicated in the regulation of life span, it was of interest to examine the possible role of the microbiome in mifepristone life span extension.

Characterization of the microbiome of laboratory *Drosophila* reveals several dozen species, including abundant *Acetobacter* and *Lactobacillus* species, and species common to humans (Ren et al., 2007; Wong et al., 2011; Broderick and Lemaitre, 2012). Bacteria are found on the surface of the fly, as well as in the fly interior and gut lumen, and the bacterial load in each location increases dramatically during aging (Ren et al., 2007; Broderick and Lemaitre, 2012). Aero-tolerance is the ability of bacteria to grow in the presence of oxygen in the air (Imlay, 2002). Obligate aerobes require oxygen for growth, whereas anaerobes do not. Aero-tolerant anaerobes are therefore species that do not require oxygen, but are able to grow in its presence. Both aerobic and aero-tolerant anaerobic species are found on the fly surface and in the fly interior and gut, and both increase in abundance dramatically during aging (Ren et al., 2007).

To study the potential role of bacteria in *Drosophila* life span, various methods have been used to reduce or eliminate the bacteria, including egg sterilization, use of autoclaved media, and treatment of flies with antibiotics (Grenier and Leulier, 2020). The commensal bacteria have been reported to have beneficial, detrimental, or no effect on life span in males and females (Brummel et al., 2004; Ren et al., 2007; Petkau et al., 2014; Clark et al., 2015; Yamada et al., 2015; Loch et al., 2017; Clark and Walker, 2018; Lee et al., 2019; Sciambra and Chtarbanova, 2021). Tert-butyl hydroperoxide is an organic alkyl-hydroperoxide oxidizing compound, often used as an antibacterial agent (Chen et al., 2021; Naguib et al., 2022). Treating young flies with low doses of tert-butyl hydroperoxide was reported to increase both male and female life span by preferentially killing *Acetobacter* species (Obata et al., 2018). Increased *Gammaproteobacteria* and *Alphaproteobacteria* and decreased *Firmicutes* have been reported to correlate with fly mortality associated with the loss of intestinal barrier integrity (Clark et al., 2015; Clark and Walker, 2018).

Here, several experiments were performed to determine whether mifepristone might interact with or target the microbiome. In the egg sterilization experiment, flies were cultured under normal conditions as well as from sterilized eggs, and life span was assayed in presence and absence of mifepristone, to determine whether effects on life span might be additive. In the methoprene experiment, flies were ones that had previously been treated as adults with the juvenile hormone analog methoprene, which shortens life span, and methoprene plus mifepristone, which rescues the shortened life span, and then frozen for subsequent analysis of bacterial DNA (Landis et al., 2021a). In the antibiotic experiment, mated females were treated with high concentrations of five individual antibiotics to determine whether any single antibiotic might mimic the longevity-promoting effects of mifepristone. For each of these three experiments, high-throughput sequencing of 16S bacterial rDNA sequences was used to determine bacterial species identity and abundance, to confirm whether the life span changes might correlate with changes in the microbiome. In addition, five species of bacteria were cultured from adult flies and grown on culture plates in the presence and absence of mifepristone, to ask whether mifepristone might exhibit any antibiotic activity *in vitro*. Finally, adult flies were maintained in the presence of high concentrations of *Enterococcus faecalis* to determine whether this bacteria species would affect life span, in the presence or absence of mifepristone.

## Materials and Methods

### *Drosophila* Strains, Culture, Drug Treatments, and Life Span Assay

*Drosophila melanogaster* were cultured at 25 °C using a standard agar/dextrose/corn meal/yeast media (Ren et al., 2009), and adult flies were passaged to fresh media every other day. *Drosophila* strains are as previously described (Landis et al., 2021a) and were obtained from the Bloomington *Drosophila* Stock Center, including y[1] w[*]; P{w[+mC] = elav-Switch.O}GSG301 strain (BDSC#43642, abbreviated *y; Elav-GS*). Virgin females with sex peptide overexpression (*w[1118]; UAS-SP/dsx-GAL4*) were generated as previously described (Tower et al., 2017). The *w[1118]* strain is the isogenized version (*w[1118]-iso; 2-iso; 3-iso*). Both the *w[1118]* strain and *y; Elav-GS* strain were previously cured of *Wolbachia* by three generations treatment with doxycycline, with confirmation using PCR and *Wolbachia*-specific primers (Ren et al., 2007; Tower et al., 2017). To generate flies for life span assays, *w[1118]* strain males were crossed to *y; Elav-GS* strain virgin females, and hybrid female progeny were collected as virgins over 24 h. These flies were either assayed as virgins or were mated for 48 h to young (one to two weeks of age) males at a ratio of 20 males to 20 females. For the sterilization experiment, the males were sterile siblings, for the antibiotic experiment, the males were siblings, and for all other experiments, the males were *w[1118]* strain. After mating, the males were removed and flies were maintained in culture vials in the presence/absence of drug, as indicated. Drugs were administered as previously described, by applying 100 μL of 10X stock solution in water, or 50 μL of 20X stock solution in ethanol, evenly to the surface of the vial, and allowing to absorb and dry overnight (Ren et al., 2009; Landis et al., 2020). Final concentration of drug in the media was calculated based on absorption into the top ∼1 ml of media, as determined by dye-absorption controls (Ren et al., 2009; Landis et al., 2020); control vials received equal volume of water or ethanol vehicle, and all vials were allowed to dry overnight. Mifepristone (RU486) was obtained from Sigma-Aldrich (cat. #M8046), and flies were treated with 200 μg/ml final concentration in the media. All flies were transferred to fresh vials every other day, and the number of dead flies was recorded. Median life span, percent change in median, log-rank *p* values, and COX proportional hazards analyses were conducted using R statistical environment v4.0.3 (Rcoreteam, 2020). Log-rank analysis was corrected for multiple comparisons using Bonferroni correction, and the *p*-value for significance at 5% error rate is indicated in the figure legends. Mann–Whitney, Kruskal–Wallis, Shapiro–Wilk, and ANOVA analyses were conducted using Prism 9, and multiple comparisons were controlled using Tukey’s correction. Flies for DNA isolation were maintained in vials in parallel to the life span assays for 12 days of drug treatment and frozen at -80°C for subsequent DNA isolation. DNA was extracted using the ZymoBIOMICS DNA Miniprep Kit (Zymo Research #D4300). Ten flies for each sample were homogenized in 200 μL lysis solution, and DNA was purified as per the manufacturer instructions. DNA concentration was measured using Nanodrop. DNA integrity was confirmed by using agarose gel electrophoresis, ethidium bromide staining, and comparison to molecular weight standards.

### Egg Sterilization

Egg sterilization was carried out essentially as previously described (Ren et al., 2007). Virgins of *y; ElavGS* strain and *w[1118]* strain males were mated in fly cages (Genesee Sci. #59–101), and eggs were collected on plates containing grape agar (Genesee Sci. #47–102) coated with a thin layer of live yeast paste. Eggs from the first overnight collection were discarded, and the eggs from the second overnight collection were used for experiments. Eggs were washed off the plates using sterile PBS and camel hair brush into a small mesh basket (Genesee Sci. #46–102). Eggs were submerged in solution of 0.25% chlorite (Clorox brand) for two minutes, then submerged in solution of 0.04% N-alkyl-N,N-dimethyl-N-benzylammonium chloride for two minutes, and then rinsed twice by submersion in sterile PBS. Eggs were transferred into 15 ml sterile tube using sterile brush and sterile PBS, allowed to settle, and excess PBS was removed. Approximately 32 μL eggs were transferred to each sterile (autoclaved) bottle of media. Adult virgin females were collected using sterile brush and pad, and mated females were generated by mating at 1:1 ratio with sterile male siblings for 48 h. Life span assays were conducted using food vials that had been surface-sterilized with 50 μL ETOH or 50 μL 4 mg/ml mifepristone in ETOH, and dried overnight.

### Antibiotic Treatment

Virgin and mated flies were generated as described previously, except the Bloomington *Drosophila* Stock Center strain used, y[1] w[*]; P{w[+mC] = elav-Switch.O}GSG301 (BDSC#43642, abbreviated *y; Elav-GS*) was not cured of *Wolbachia* prior to use. Antibiotics were administered at the following final concentrations in the vials: doxycycline, 640 μg/ml (Sigma #D9891); ampicillin, sodium salt, 1280 μg/ml (VWR # 97061-442); kanamycin sulfate, 2 mg/ml (Shelton Sci. #IB02120); streptomycin sulfate, 2 mg/ml (Shelton Sci. IB02180); and control vials received purified water vehicle. Life span was assayed as abovementioned, with modification that the first two weeks were censored to remove some early deaths attributed to handling.

### Methoprene Treatment

The flies were generated in parallel with flies previously assayed for life span and were previously assayed for food intake (Landis et al., 2021a); see previous Table S11 for the tabular presentation of life span data and previous Figure S3F,G for food intake assays. The life span data were re-analyzed and plotted using updated version R statistical environment v4.0.3. Virgin female flies (progeny of cross *w[1118] x y; Elav-GS*) were maintained on control media, 200 μg/ml mifepristone, 200 μg/ml methoprene (Cayman #16807), or 200 μg/ml methoprene plus 200 μg/ml mifepristone. Flies were frozen at −80°C after 16 days drug treatment and used for DNA isolation and analysis.

### Zymo Research 16S Sequencing Analysis

Details were adapted from manufacturer’s descriptions: DNA samples were prepared using Quick-16S™ NGS Library Prep Kit (Zymo Research, Irvine, CA). The primer set used was Quick-16S™ Primer Set V3-V4 (Zymo Research, Irvine, CA), which amplifies a hyper-variable region of the bacterial rDNA to distinguish bacterial species. PCR reactions were performed in real-time PCR machines to control cycles and limit chimera formation. PCR products were quantified using qPCR fluorescence readings and pooled based on equal molarity. The pooled library was treated with Select-a-Size DNA Clean & Concentrator™ (Zymo Research, Irvine, CA) and quantified with TapeStation^®^ (Agilent Technologies, Santa Clara, CA) and Qubit^®^ (Thermo Fisher Scientific, Waltham, WA). Sequencing was conducted on Illumina^®^ MiSeq™ with a v3 reagent kit (600 cycles) and with 10% PhiX spike-in. Unique amplicon sequences were inferred from raw reads and chimeric sequences removed using DADA2 pipeline (Callahan et al., 2016). Taxonomy assignment was performed using Uclust from Qiime v.1.9.1. and the Zymo Research Database, a 16S database that is internally designed and curated, as reference. For absolute abundance quantification, a quantitative real-time PCR was set up with a standard curve made with plasmid DNA containing one copy of the 16S gene and one copy of the fungal ITS2 region, prepared in ten-fold serial dilutions. The standard curve was used to calculate the number of gene copies for each sample. The number of genome copies was calculated assuming four 16S gene copies per genome. Data are expressed as number of genome copies per fly. For each of the three experiments (sterilization, antibiotic, and methoprene), the total reads were calculated for each species across all the samples. These species totals were averaged, and any species or class that had greater than nine times the average was defined as “outlier”. For the sterilization experiment, three outlier species were found: *Lactobacillus* (204 times average totals), *Komagataeibacter medellinensis-oboediens-xylinus* (116 times average totals), and *Acetobacter persici* (55 times average totals); two outlier classes were found: *Bacilli* (14 times average totals) and *Alphaproteobacteria* (12 times average totals). For the methoprene experiment, two outlier species were found: *Lactobacillus* (105 times average totals) and *Komagataeibacter medellinensis-oboediens-xylinus* (36 times average totals) and one outlier class was found: *Bacilli* (11 times average totals). For the antibiotic experiment, two outlier species were found: *Lactobacillus* (232 times average totals) and *Komagataeibacter medellinensis-oboediens-xylinus* (18 times average totals) and one outlier class was found: *Bacilli* (18 times average totals). For statistical comparisons, any species that had less than ten average reads in either control or treatment group was excluded. Data are presented for species and class, both including and excluding outliers, as indicated in figure legends. Determination of normal distribution of data was conducted using Shapiro–Wilk test. For all classes, and the great majority of species comparisons, the data were not normally distributed, and nonparametric Mann–Whitney and Kruskal–Wallis test results are presented. For the species comparison with normally distributed data, the unpaired, two-sided *t*-test result is presented. Certain species names are hereafter abbreviated as follows: *Komagataeibacter medellinensis-oboediens-xylinus, Komagataeibacter m; Staphylococcus capitis-caprae-epidermidis, Staphylococcus c;* Corynebacterium pseudogenitalium-tuberculostearicum, Corynebacterium p; and *Staphylococcus epidermidis-hominis, Staphylococcus e*. The raw sequencing data have been deposited at the NCBI SRA database, reference: PRJNA835902.

### Bacterial Isolation and Sequencing

Middle-aged (∼40 days old) mated females, progeny of *w[1118]* x *y; ElavGS* cross, were homogenized in PBS (ten flies per extraction), and dilutions of the extracts were plated on nutrient agar plates and on MRS plates, as previously described (Ren et al., 2007). Single colonies were picked and re-plated, and single colonies were again picked and used for further culture and for sequencing. DNA was purified from each of five unique isolates and sequenced using primers specific for 16S region (Ren et al., 2007). Sequences were compared to GenBank using BLASTN, and this identified species *Lactobacillus plantarum*, *Acetobacter sicerae*, *Enterococcus faecalis*, *Paenibacillus glucanolyticus* and *Serratia rubidaea*. Sequences are deposited at GenBank (SUB11189544).

## Results

### Egg Sterilization and Mifepristone Have Additive Benefit for Life Span

**FIGURE 1:**
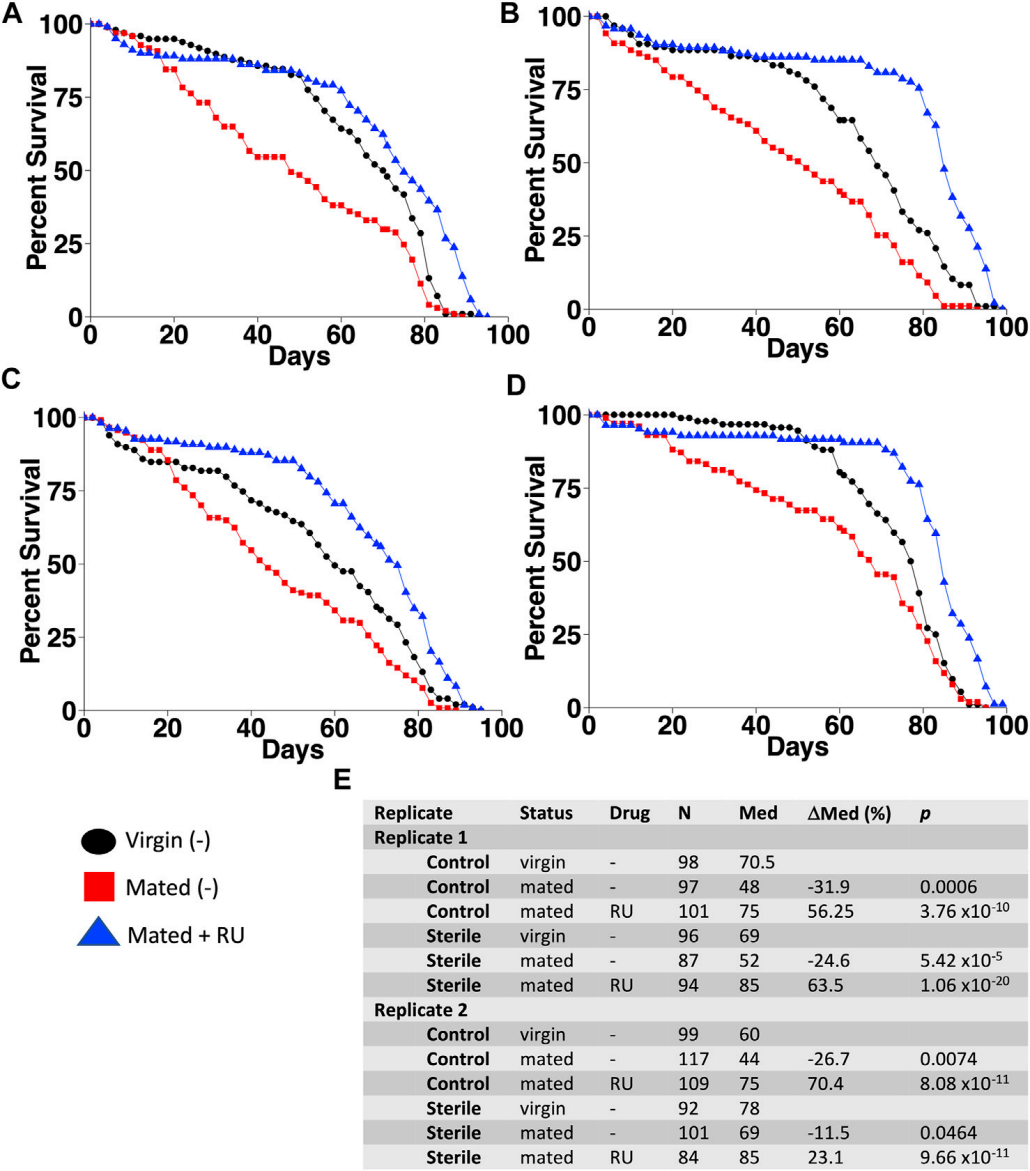
Additive life span benefit from egg sterilization and mifepristone. Life span was assayed in virgin females, mated females, and mated females plus mifepristone/RU486 (RU), using control culture conditions **(A,C)** and using egg sterilization and autoclaved media **(B,D). (A)** Control conditions, experiment 1. **(B)** Sterile conditions, experiment 1. **(C)** Control conditions, experiment 2. **(D)** Sterile conditions, experiment 2. **(E)** Statistical summary. For each experiment, mated is compared to virgin, and mated plus drug is compared to mated. Statistical test: log-rank; *p-*value for significance with two comparisons is 0.025.

To investigate the potential interactions between mifepristone and the microbiome, life span was assayed for flies that had been grown from sterilized eggs on autoclaved media and compared to flies grown in parallel under normal, nonsterile conditions (Figure 1). In the flies grown under sterile conditions, the transfer of bacteria from parents to offspring is eliminated. Virgin female adults were collected using a sterile brush and fly pad, and mated females were generated by mating with sterile male siblings for 48 h. Life span was assayed for virgins, mated females, and mated females plus mifepristone, in replicate experiments. Mating decreased life span, and mifepristone dramatically increased the life span of the mated females, under both normal and sterile conditions (Figure 1). Notably, there was an additive life span benefit from sterilization and mifepristone. Under control conditions, the mifepristone-treated mated females had a median life span of 75 days in each replicate experiment, whereas under sterile conditions, the mifepristone-treated mated females had a median life span of 85 days in each replicate experiment. COX proportional hazards analysis confirmed the significant effects of sterile condition, mifepristone, and the sterile condition/mifepristone interaction (Supplementary Table S1). In addition, COX proportional hazard analysis confirmed that the control groups were not different from each, and that the sterile groups were not different from each other, but that the sterile groups lived longer than the control groups, as expected (Supplementary Table S1).

### Mifepristone Does Not Detectably Decrease Adult Fly Bacterial Load

**FIGURE 2:**
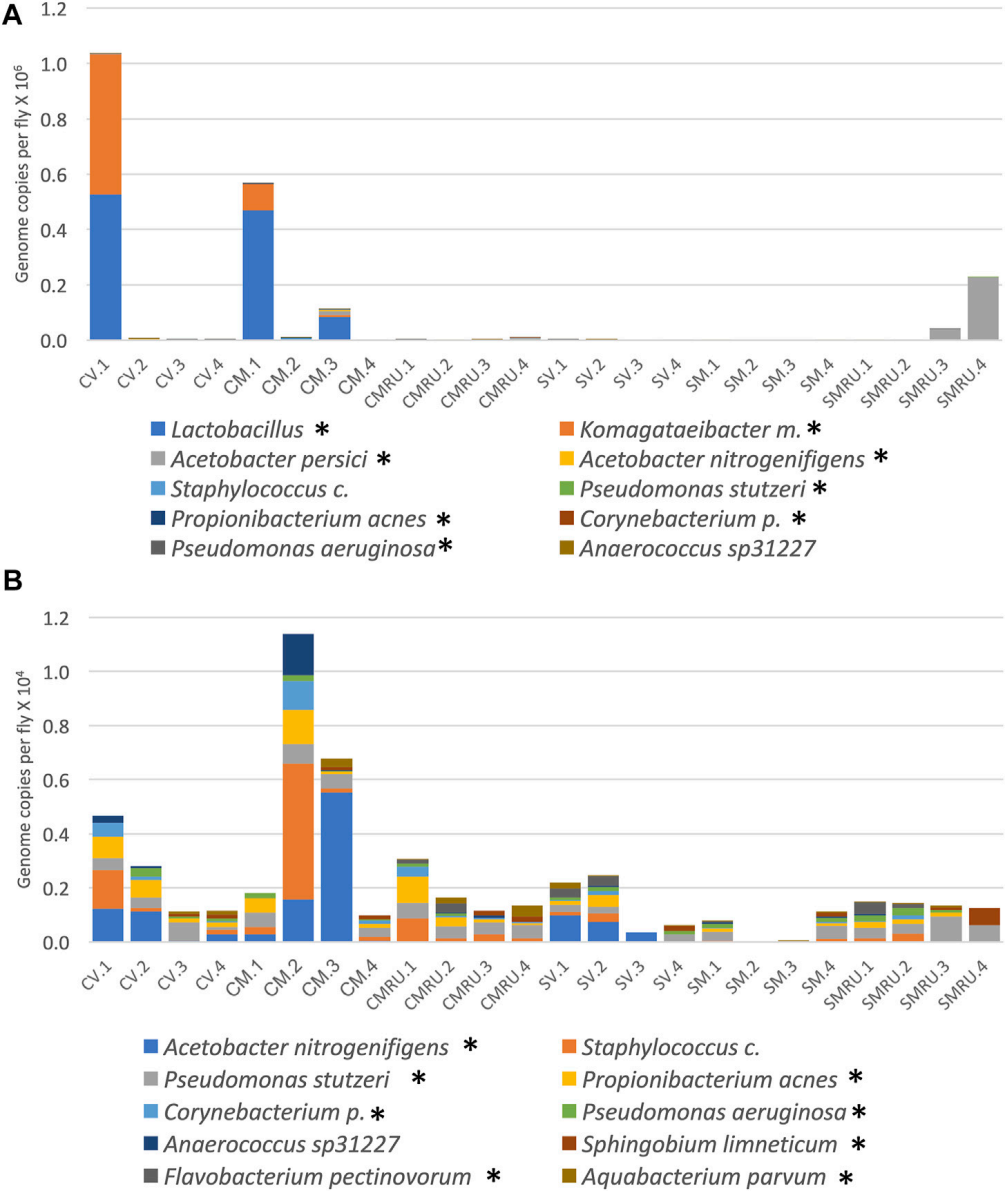
Effect of sterilization and mifepristone on adult female bacterial species and identification of outliers. **(A)** Top 10 species, including outliers. **(B)** Top 10 species, excluding outliers. CV, control virgins; CM, control mated; CMRU, control mated plus mifepristone/RU486; SV, sterile virgins; SM, sterile mated; and SMRU, sterile mated plus mifepristone/RU486. Species and classes reported to be aero-tolerant are indicated with asterisk.

**FIGURE 3:**
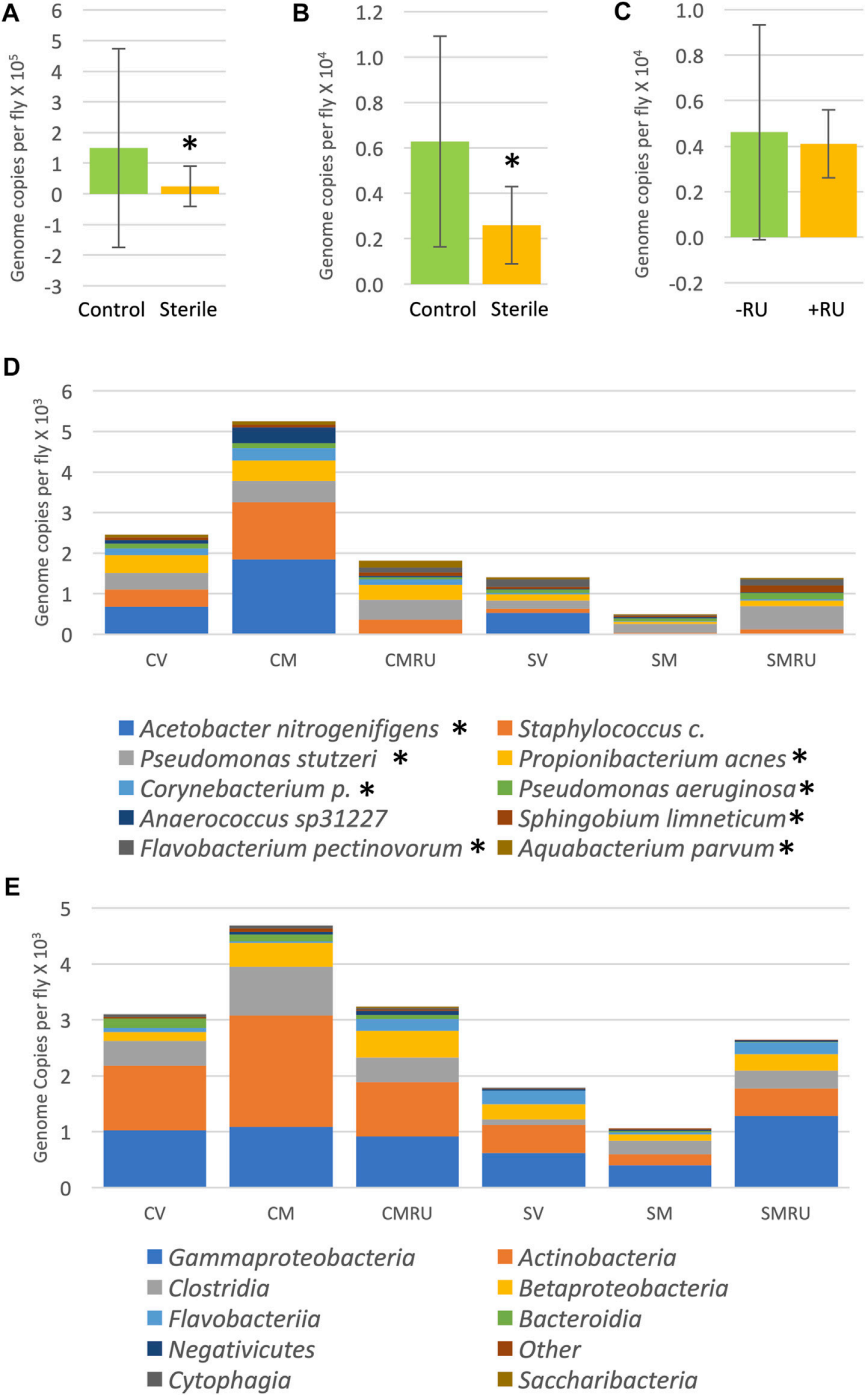
Effect of egg sterilization and mifepristone on adult female bacterial species and classes. **(A)** Total bacterial load of control flies compared to sterile condition flies, including outliers. Mann–Whitney *p* = 0.0145 (indicated with asterisk). **(B)** Total bacterial load of control flies compared to sterile condition flies, excluding outliers. Mann–Whitney *p* = 0.0173 (indicated with asterisk). **(C)** Total bacterial load of nontreated flies (−RU) compared to mifepristone (RU486)–treated flies (+RU), excluding outliers. Mann–Whitney *p* = 0.3286. **(D)** Average top ten species (excluding outliers). **(E)** Average top ten classes (excluding outliers). CV, control virgins; CM, control mated; CMRU, control mated plus mifepristone/RU486; SV, sterile virgins; SM, sterile mated; and SMRU, sterile mated plus mifepristone/RU486. Species and classes reported to be aero-tolerant are indicated with asterisk.

To investigate the effects of egg sterilization and mifepristone treatment on the adult fly microbiome, the bacterial species diversity and abundance were assayed using high-throughput sequencing. The flies were assayed after 12 days of mifepristone treatment, which is when life span curves for control and mifepristone-treated flies have begun to diverge (Figure 1). Four replicate samples were sequenced for each of the groups: virgin females, mated females, and mated females plus mifepristone, for both the sterile condition and control condition flies. Each replicate came from a different culture vial maintained in parallel with vials used for life span assay. Species analysis revealed that five out of 24 samples had exceptional growth of one or two species, *Lactobacillus* and *Komagataeibacter medellinensis-oboediens-xylinus* (hereafter abbreviated *Komagataeibacter m.*)*,* or *Acetobacter persici* (Figure 2A)*.* Note that in the Zymo Research annotation, all *Lactobacillus* species are grouped together under heading *Lactobacillus*. Each of these three outlier species had an average abundance across all samples that was nine times greater than any other species. The observation of outliers is consistent with previous reports of dramatic variation in bacterial species abundance between replicate vials of *Drosophila*, as well as between individual flies (Ren et al., 2007; Broderick et al., 2014; Duneau et al., 2017). As a result, there were also two outlier classes, *Bacilli* and *Alphaproteobacteria,* that likewise had an average abundance across all samples that was nine times greater than any other class. Plotting the data omitting the three outlier species enabled the visualization of top ten species diversity across the samples and suggested reduced bacterial load in the sterile condition flies (Figure 2B). Consistent with that observation, the comparison of total bacterial load between the control-condition and sterile-condition flies revealed a significant reduction in the sterile-condition flies, when outliers were included in the analysis (Figure 3A), and when outliers were omitted from the analysis (Figure 3B). In contrast, when mifepristone-treated flies were compared to untreated flies, no significant difference in total bacterial load was detected (Figure 3C). The sterile condition caused reduced abundance of six species and four classes relative to control-condition flies (Figure 3D,E; Table 1). In contrast, mifepristone treatment did not cause any detectable decreases and was instead associated with increased abundance of three species (Figure 3D,E; Table 1). We note that only one of these species, *Pseudomonas stutzeri,* is among the top ten most abundant species observed in the experiment (Figure 3D). The observation that mifepristone was associated with increased abundance of three species (Table 1), but not a significantly increased total bacterial load (Figure 3C), is likely because the large variability in total bacterial load across samples masks the change due to these mostly minor species.

**TABLE 1:** Bacterial species changes due to egg sterilization and mifepristone. UD, undefined decrease (treatment value equal to zero); UI, undefined increase (control value equal to zero). Statistical test: Mann–Whitney nonparametric test. For mifepristone species *Pseudomonas stutzeri,* the *p-*value is presented for unpaired, two-sided *t*-test.

Sterile Condition Species		Fold Change	*p-*Value
—	*Peptoniphilus grossensis*	UD	0.0046
	*Propionibacterium acnes*	0.25	0.0081
	*Anaerococcus prevotii-tetradius*	0.19	0.0182
	*Cardinium* endosymbiont	0.043	0.0207
	*Pseudomonas stutzeri*	0.70	0.0447
	*Corynebacterium accolens*	0.11	0.0494
**Sterile condition classes**			
—	*Bacteroidia*	0.14	0.0006
	*Bacilli*	0.04	0.0007
	*Actinobacteria*	0.30	0.0023
	*Cytophagia*	0.17	0.0207
**Mifepristone species**			
—	*Lachnoclostridium sp32445*	UI	0.0277
	*Eubacterium rectal*	4.0	0.0318
	*Pseudomonas stutzeri*	1.6	0.0407

**FIGURE 4:**
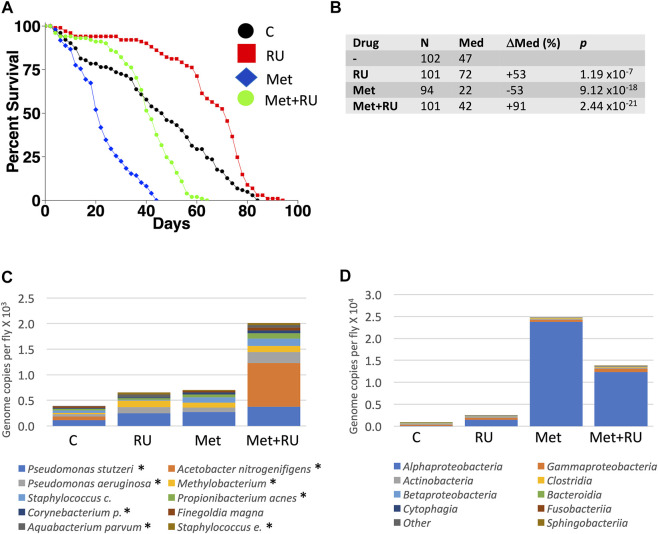
Effect of methoprene and mifepristone on virgin female life span, bacterial species, and classes. **(A)** Survival curves. C, minus-drug control; RU, mifepristone; and Met, methoprene. Data are replotted from Landis et al. (2021a). **(B)** Life span statistical summary. Percent change in median life span was calculated by comparing RU to C, and by comparing Met to C, and by comparing Met + RU to Met. Statistical test: log-rank; *p-*value for significance with three comparisons is 0.017. **(C)** Average top ten species (excluding outliers). **(D)** Average top ten classes (excluding outliers). C, control; RU, mifepristone/RU486; and Met, methoprene. For both species and classes, no statistically significant differences were detected between groups using Kruskal–Wallis test. Species and classes reported to be aero-tolerant are indicated with asterisk.

The juvenile hormone analog methoprene recapitulates many of the effects of mating in virgin female flies, including decreased median life span (Figure 4A; data were replotted from Landis et al. (2021a)). Mifepristone doubles the life span of methoprene-treated virgins, (Figure 4A), while simultaneously doubling food intake (Landis et al., 2021a). To determine whether mifepristone would be associated with altered microbiome under these conditions, bacterial species abundance was assayed in three replicate samples for each of the four groups of virgin females, at day 16 of drug treatment: control, mifepristone-treated, methoprene-treated, and mifepristone plus methoprene-treated. Two outlier species were found, *Lactobacillus* and *Komagataeibacter m.*, and one outlier class was found, *Bacilli* (Supplementary Figure S1A,B). Mifepristone had no statistically significant effect on total bacterial load (Supplementary Figure S1C,D). Plotting the top ten species and top ten classes suggests some variation across groups (Figure 4C,D); however, no significant differences in bacterial species or class abundance between the groups was found using Kruskal–Wallis test. In summary, in multiple experiments where mifepristone increased life span, the bacterial species and classes were either unchanged or sometimes increased.

### Mifepristone Has No Detectable Antibacterial Effects *In Vitro*

**FIGURE 5:**
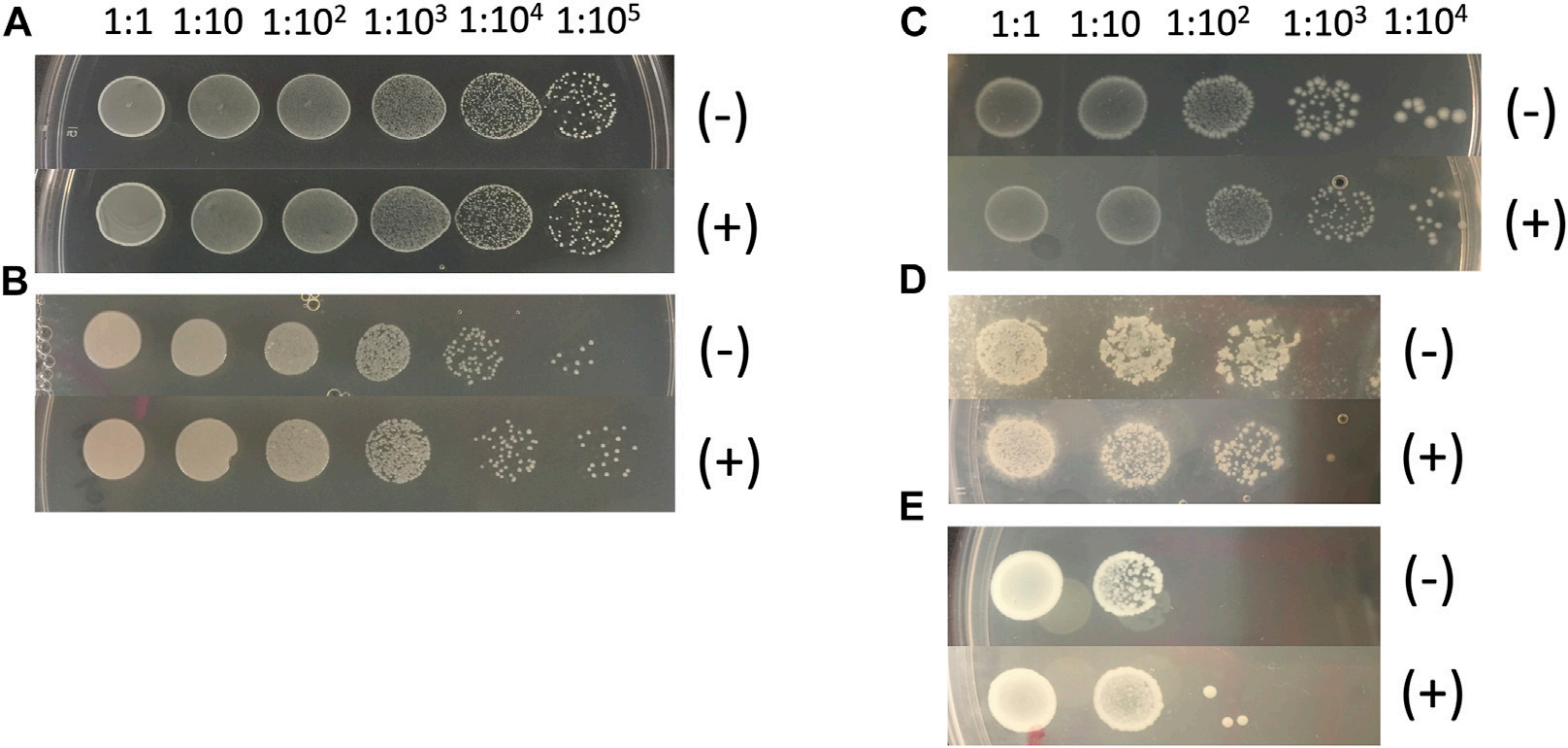
Mifepristone has no detectable antibacterial properties *in vitro*. Five abundant bacterial species were purified from single colonies. The colonies were produced by plating extracts of middle-age mated females, using the strain with the largest magnitude response to mating and to mifepristone (progeny of *w[1118] X y;Elav-GS*). Bacterial species identity was determined by sequencing bacterial 16S DNA. Over-night liquid cultures of the indicated bacterial species were serially diluted as indicated, and 2 μL aliquots were spotted onto control plates (-), and plates adjusted to 200 μg/ml final concentration mifepristone (+). **(A)** *Lactobacillus plantarum*. **(B)** *Acetobacter sicerae.* **(C)** *Enterococcus faecalis*. **(D)** *Paenibacillus glucanolyticus*. **(E)** *Serratia rubidaea*.

Bacteria were cultured from extracts of middle-aged, mated female flies, and DNA was purified from each of five unique isolates. 16S sequences were compared to GenBank, and identified species *Lactobacillus plantarum*, *Acetobacter sicerae*, *Enterococcus faecalis*, *Paenibacillus glucanolyticus* and *Serratia rubidaea*. Because microbial load increases with age (Ren et al., 2007), middle-aged mated females were used to increase the abundance of bacteria for possible culture; however, we note that several of these species have also been found in flies that were less than ten days old (*Lactobacillus plantarum* and *Acetobacter sicerae*) and less than twenty days old (*Enterococcus faecalis and Paenibacillus glucanolyticus*) by culturing and/or 16S sequencing (Storelli et al., 2011; Feltzin et al., 2019; Lee et al., 2019). Each of these five species was grown in overnight liquid cultures, and dilutions of the cultures were plated on control plates and on plates supplemented with 200 μg/ml mifepristone. The presence of mifepristone had no detectable negative effect on growth, indicating that mifepristone does not have a direct antibiotic effect on these five species (Figure 5).

### Individual Antibiotics Do Not Mimic the Life Span Benefit of Mifepristone

**FIGURE 6:**
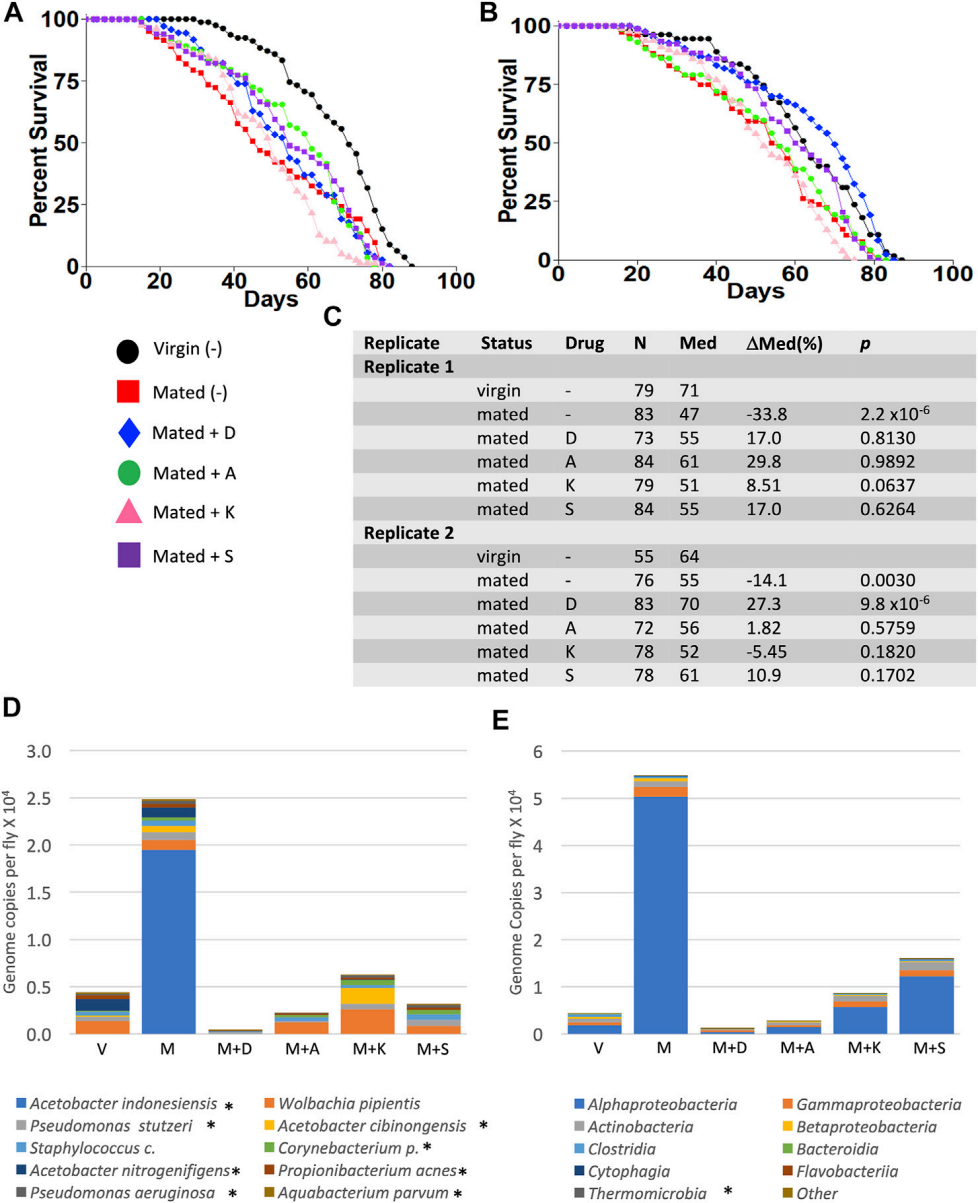
Effect of high-concentration antibiotics on mated female life span and microbial load. Life span was assayed in replicate experiments, for virgin females (V), mated females (M), and mated females (M), and mated females treated with doxycycline (D), ampicillin (A), kanamycin (K), and streptomycin (S). **(A)** Experiment 1. **(B)** Experiment 2. **(C)** Life span statistical summary. Percent change in median life span was calculated by comparing mated to virgin, and by comparing antibiotic-treated mated to mated. Statistical test: log-rank; *p-*value for significance with five comparisons is 0.01. **(D)** Average bacterial species (excluding outliers). **(E)** Average bacterial classes (excluding outliers). Species and classes reported to be aero-tolerant are indicated with asterisk.

Experiments were conducted to determine whether any single antibiotic could recapitulate the life span effects of mifepristone in adult mated females. Mated females were assayed for life span in the presence of high concentrations of the antibiotics doxycycline (640 μg/ml), ampicillin (1280 μg/ml), kanamycin (2 mg/ml), and streptomycin (2 mg/ml), in replicate experiments. No significant benefits were observed for ampicillin, kanamycin, or streptomycin. Doxycycline had a significant benefit in experiment 2 (+27%), but no significant benefit in experiment 1 (Figure 6). Bacterial species abundance was assayed in three replicate samples for each of the five groups of mated females: no drug, doxycycline, ampicillin, kanamycin, and streptomycin. Two outlier species were found: *Lactobacillus* and *Komagataeibacter m.,* and one outlier class was found: *Bacilli* (Supplementary Figure S2A,B). Plotting the top ten species and top ten classes suggests some variation across groups (Figure 6D,E); however, no statistically significant differences in bacterial species or class abundance were found using Kruskal–Wallis test.

### Supplementation of Media With *Enterococcus faecalis* Has No Effect on Life Span in the Presence or Absence of Mifepristone

**FIGURE 7:**
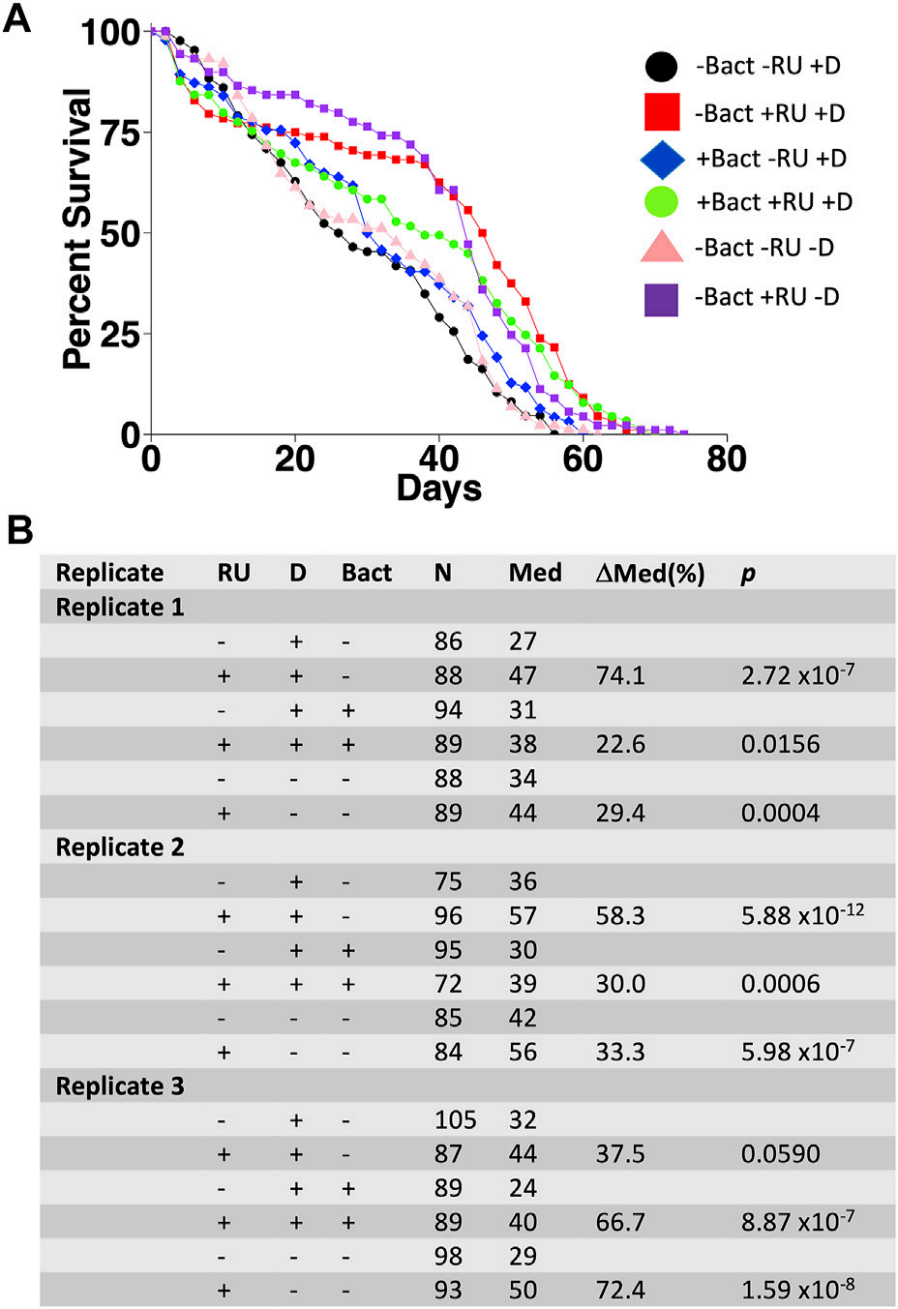
Effect of *Enterococcus faecalis* supplementation on adult female life span. Flies with constitutive expression of sex peptide (genotype *w[1118];dsx-GAL4/+; UAS-SP/+*) were grown on media in absence (-D) and presence (+D) of doxycycline. Virgin females were collected and assayed for life span in absence (-Bact) or presence (+Bact) of added *Enterococcus faecalis*, and in absence (−RU) or presence (+RU) of 200 μg/ml mifepristone/RU486, in three replicate experiments. **(A)** Survival curves for experiment 1. **(B)** Statistical summary for experiments 1–3. For each experiment, +RU is compared to −RU. Statistical test: log-rank; *p-*value for significance with one comparisons is 0.05.

*Enterococcus faecalis* is normally found in the human gastrointestinal tract and is often found associated with laboratory *Drosophila* (Cox and Gilmore, 2007; Broderick and Lemaitre, 2012; Loch et al., 2017) (this study). Of the five species described above that were cultured from mated females, *Enterococcus faecalis* was chosen for further study because of its ease of culture, frequent association with *Drosophila*, and its relevance to human health (Kao and Kline, 2019; Dadashi et al., 2021). To determine whether *Enterococcus faecalis* might affect life span and interact with mifepristone *in vivo*, adult females were assayed for life span using control media and media supplemented with *Enterococcus faecalis* in the presence or absence of mifepristone. To avoid bacteria that might be introduced by mating, the females were virgins with transgenic expression of sex peptide, which recapitulates the negative effect on life span caused by mating (Tower et al., 2017). Flies were grown both in normal media (-D) and in media supplemented with doxycycline (+D), in effort to reduce endogenous bacterial load prior to the introduction of *Enterococcus faecalis*. Vials used in the life span assay were supplemented with *Enterococcus faecalis* by adding 100 μL of fresh overnight culture (∼5 × 10^6^ CFU) to the surface of the media 48 h prior to use. Life span was assayed in three replicate experiments, and mifepristone increased life span in each experiment (Figure 7). COX proportional hazards analysis confirmed the significant effect of mifepristone and showed no significant effect of *Enterococcus faecalis* and no significant *Enterococcus faecalis*/mifepristone interaction (Supplementary Table S1). Interestingly, life span varied between replicate experiments, and COX proportional hazards analysis confirmed a significant difference between replicates (Supplementary Table S1). The possible reasons for this variability are currently not clear.

## Discussion

### Mifepristone Has No Detectable Antibiotic Effect *In Vivo* or *In Vitro*

In this study, several approaches were used to investigate possible interactions between mifepristone and the *Drosophila* bacterial microbiome in the context of life span regulation. Egg sterilization prevents transfer of bacteria from parents to offspring, and this resulted in increased female life span, consistent with previous reports (Clark et al., 2015; Lee et al., 2019). Treatment with 200 μg/ml mifepristone further increased female life span, consistent with the conclusion that sterilization and mifepristone increase life span through different targets. High-throughput sequencing of bacterial 16S sequences confirmed that egg sterilization reduced total bacterial abundance and reduced the abundance of six specific bacterial species (Table 1). These species are therefore candidates for ones that might be causative in reducing life span. By contrast, mifepristone treatment was not associated with any decreases, and instead was associated with increased abundance of several species, indicating that mifepristone does not have antibacterial activity *in vivo*. The juvenile hormone analog methoprene recapitulates many aspects of mating, including reducing female life span (Yamamoto et al., 2013; Landis et al., 2021a). Mifepristone rescues the negative effects of methoprene, and again this life span extension was not associated with any decrease in bacterial sequences, further supporting the conclusion that mifepristone does not have antibacterial effects *in vivo*. The total bacterial abundance for the virgin control flies in the methoprene experiment (∼0.8 × 10^3^, Supplementary Figure S1D) is lower than that for virgin female flies from the egg sterilization experiment (∼0.5 × 10^4^) and the antibiotic experiment (∼0.4 × 10^4^, Supplementary Figure S2D), and this may be related to the >1 year separation in time between the experiments (Landis et al., 2021a). The strains used for the egg sterilization and methoprene experiments were negative for the endosymbiotic bacteria such as *Wolbachia*, thereby confirming that mifepristone life span increase is independent of *Wolbachia* (Maistrenko et al., 2016).

Treating adult female flies with high concentrations of the individual antibiotics doxycycline, ampicillin, kanamycin, or streptomycin did not have a consistent benefit for female life span, indicating that these antibiotics cannot reproduce the life span benefits of mifepristone. Because egg sterilization reduced bacterial abundance and increased life span, but the individual antibiotics did not, we conclude that the antibiotics and concentrations tested are not sufficient to target the relevant bacterial species. Finally, 200 μg/ml mifepristone had no detectable effect on the growth of five bacterial species cultured from adult females (*Lactobacillus plantarum*, *Acetobacter sicerae*, *Enterococcus faecalis*, *Paenibacillus glucanolyticus,* and *Serratia rubidaea*)*,* indicating that mifepristone has no direct antibiotic activity *in vitro*, at least with these five species tested. Consistent with that conclusion, Loch et al. (Loch et al., 2017) reported that 100 μg/ml mifepristone had no negative effect on the growth of *Pseudomonas entomophila,* or on growth of total bacteria cultured from dissected female fly midguts, using LB media or Ace (*Acetobacter*-selective) media plates. Taken together, these data support the conclusion that mifepristone does not have antibacterial activity *in vitro* or *in vivo*, and therefore that the life span–promoting effects of mifepristone are not due to antibacterial activity.

### Outlier Species and Vial Variability

In each of the bacterial 16S sequencing analyses, a small subset of vials had exceptional growth of two or three species, *Lactobacillus*, *Komagataeibacter m.,* and *Acteobacter persici.* The observation of outliers is consistent with previous reports of dramatic variation in bacterial species abundance between replicate vials of *Drosophila*, as well as between individual flies (Ren et al., 2007; Broderick et al., 2014; Duneau et al., 2017). Other groups have also reported that *Lactobacillus*, *Komagataeibacter m.,* and *Acetobacter persici* are the dominant species in young laboratory *Drosophila* (Lee et al., 2019). Whereas certain bacterial species have been reported to stably colonize the fly gut (Pais et al., 2018), the maintenance of the majority of *Acetobacter* and *Lactobacillus* species in the fly depends on continued ingestion of the bacteria, and correspondingly the bacterial load inside the fly is generally proportional to the load on the food surface (Ren et al., 2007; Blum et al., 2013). The fact that outlier species were observed in only a small subset of vials may be a result of sampling at relatively young age (14 days), before significant age-associated expansion has occurred. Because these outlier species were limited to very few vials, they did not correlate with life span, nor were they detectably altered by mifepristone or antibiotic treatments.

### Comparison to Previous Studies of Mifepristone Effects

A previous experiment indicated that mated females had an increase in aero-tolerant species that could grow on nutrient agar plates, and that these were reduced in mifepristone-treated flies (Tower et al., 2017); however, in the present experiments no decreases were observed using 16S sequencing, and the majority of mated female samples had zero colonies when extracts were plated on nutrient agar plates (data not shown). One possibility for the difference in results might be variability in outlier species identity or abundance in the previous study. Previous transcriptomics analysis and assay of an antimicrobial peptide (AMP) gene transgenic reporter (*Drosocin*-GFP) showed an increase in AMP gene expression due to mating that was reduced in mifepristone-treated flies (Tower et al., 2017). While that observation could potentially be interpreted to result from altered bacterial load, an alternative interpretation that is consistent with the present data is mifepristone acts to reduce the innate immune response. Indeed, chronic activation of innate immune response is associated with reduced life span in both *Drosophila* and humans (Franceschi et al., 2018; Garschall and Flatt, 2018). Identification of mifepristone receptors and their potential relationship with the innate immune response will be an important area for future research.

### Variable Effects of Doxycycline on Mated Female Life Span

In these experiments, doxycycline treatment significantly increased mated female life span in only one of the two replicate experiments, and no significant effect of doxycycline on microbial load was detected using 16S RNA sequencing. Similarly, we have previously reported that doxycycline had an inconsistent effect on mated female life span between replicate experiments (Tower et al., 2017). One possibility is that doxycycline inhibits the growth of some life span–shortening bacterial species that is only variably present in the fly cohorts, and that is not detected by sequencing. Because the strain used for the antibiotics experiment was positive for *Wolbachia*, it is conceivable that the variable effect of doxycycline on life span was a result of reducing *Wolbachia* (Maistrenko et al., 2016); however, no significant reduction in *Wolbachia* abundance was detected by 16S sequencing. Another possibility is that doxycycline increases life span through a different mechanism, such as its reported ability to inhibit mitochondrial translation and FA oxidation (Freneaux et al., 1988; Curtis et al., 2007; Moullan et al., 2015; Dijk et al., 2020).

### Potential Role of Bacteria in Mifepristone Inhibition of Midgut Hypertrophy

Mating and male SP cause midgut hypertrophy in the mated female, and this hypertrophy was prevented by feeding the mated females mifepristone, for 3/3 genotypes tested (Landis et al., 2021b). Other studies have reported that the presence of bacteria stimulates midgut cell proliferation and increases midgut size relative to axenic flies (Broderick et al., 2014); however, the possible role of bacteria in mating-induced midgut hypertrophy is unclear. Because mifepristone was associated with increases in several bacterial species as opposed to a decrease, this suggests the ability of mifepristone to block midgut hypertrophy is not due to an antibiotic effect. A high yeast and low sugar diet is also reported to stimulate midgut hypertrophy, independent of cell division (Bonfini et al., 2021). Because mifepristone decreases midgut size coincident with decreasing food intake (Landis et al., 2021b), and can increase the abundance of specific bacterial species that might potentially alter nutrient availability (this study), one possibility consistent with the present results is that mifepristone blocks midgut hypertrophy by altering the absorption of one or more nutrients. Alternatively, mifepristone may act to inhibit midgut hypertrophy through a mechanism that is independent of bacterial and dietary signals, and this will also be an interesting area for future research.

### Mifepristone Life Span Extension Does Not Consistently Correlate With Bacterial Abundance

Whereas mifepristone treatment was not associated with any detectable decreases in bacterial species abundance, three species did show increased abundance upon mifepristone treatment in the egg sterilization experiment, *Lachnoclostridium sp32445*, *Eubacterium rectal,* and *Pseudomonas stutzeri* (Table 1). Conceivably, one or more of these increased species could be contributing to the increase in life span. However, *Pseudomonas stutzeri* was also reduced by egg sterilization and was therefore not consistently correlated with life span. In addition, no change in bacterial species abundance was observed when life span was increased by mifepristone in the methoprene experiment, indicating that there is no consistent correlation between bacterial abundance and mifepristone life span extension across the various experiments and conditions. Supplementing the media with high concentrations of *Enterococcus faecalis* had no effect on life span in presence or absence of mifepristone. Taken together, the data indicate that mifepristone life span extension is not associated with any detectable decrease in bacterial abundance. However, we cannot rule out the possibility that mifepristone might sometimes be associated with an increase in one or more beneficial species. Current candidates for mifepristone targets include *Drosophila* PPARγ homologs (*Eip75B and Eip78C*), the *estrogen-related receptor* (*ERR*), *Hr96,* and other nuclear hormone receptors (Landis et al., 2021a; Landis et al., 2021b; Ma et al., 2021; Praggastis et al., 2021). Given the evidence for conserved benefits of mifepristone across species, it will be of interest in the future to further analyze mifepristone mechanism(s).

## Conclusion

Mifepristone is a drug with a long record of safe use in humans for birth control and for the treatment of Cushing’s disease, and it has recently become of additional interest because of its anticancer, antiobesity, and antidiabetic effects (Gubbi et al., 2021; Llaguno-Munive et al., 2021). Mifepristone also shows beneficial metabolic effects in female *Drosophila*, including decreased lipid levels and increased life span on normal and high-fat diets (Landis et al., 2021a; Landis et al., 2021b). Here, we show that the benefits of mifepristone in female *Drosophila* are not associated with any detectable antibacterial activity, which may have relevance for our understanding of the mechanisms for mifepristone benefits in humans.

## Data Availability

The datasets presented in this study can be found in online repositories. The names of the repository/repositories and accession number(s) can be found below: NCBI BioProject - PRJNA835902.
