# Supplementary material for: Mifepristone Increases Life Span in Female Drosophila Without Detectable Antibacterial Activity
Source: Front Aging. 2022 Jul 22;3:924957. doi: 10.3389/fragi.2022.924957 (PMC9354577; doi:10.3389/fragi.2022.924957)
Supplement: Supplementary file 1 [file DataSheet1.PDF]

Mifepristone increases life span in female *Drosophila* without detectable antibacterial activity

Gary N. Landis, Luke Riggan, Hans S. Bell, William Vu, Tianyi Wang, Ina Wang, Felicia I. Tejawinata, Sebastian Ko, John Tower

SUPPLEMENTAL MATERIALS

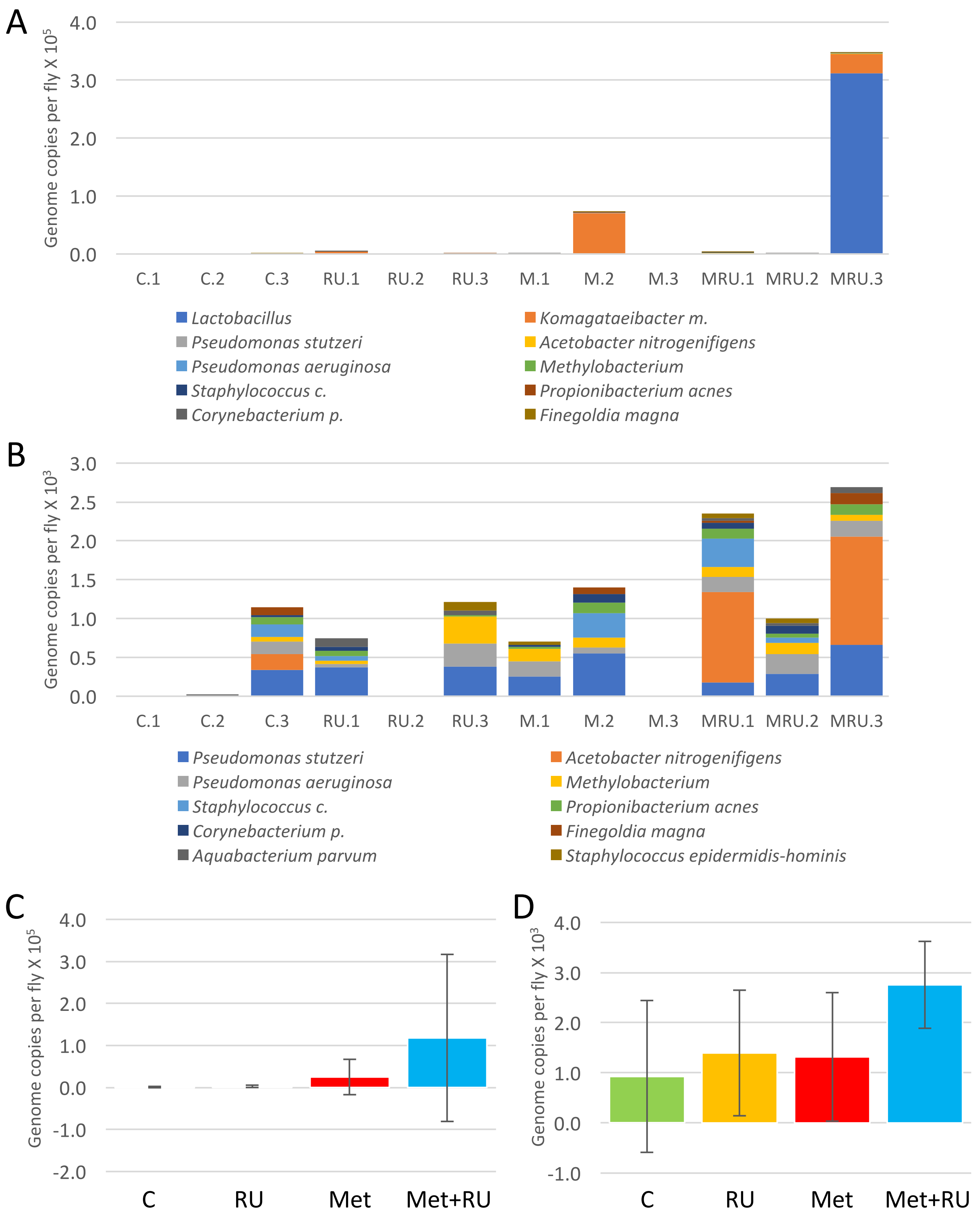

FIGURE S1. Effect of methoprene and mifepristone on virgin female bacterial load. **(A)** Top 10 species including outliers. **(B)** Top 10 species excluding outliers. **(C)** Average total bacteria including outliers. **(D)** Average total bacteria excluding outliers. No statistically significant differences were detected between groups by Kruskal-Wallis test, for species, class or total bacteria.

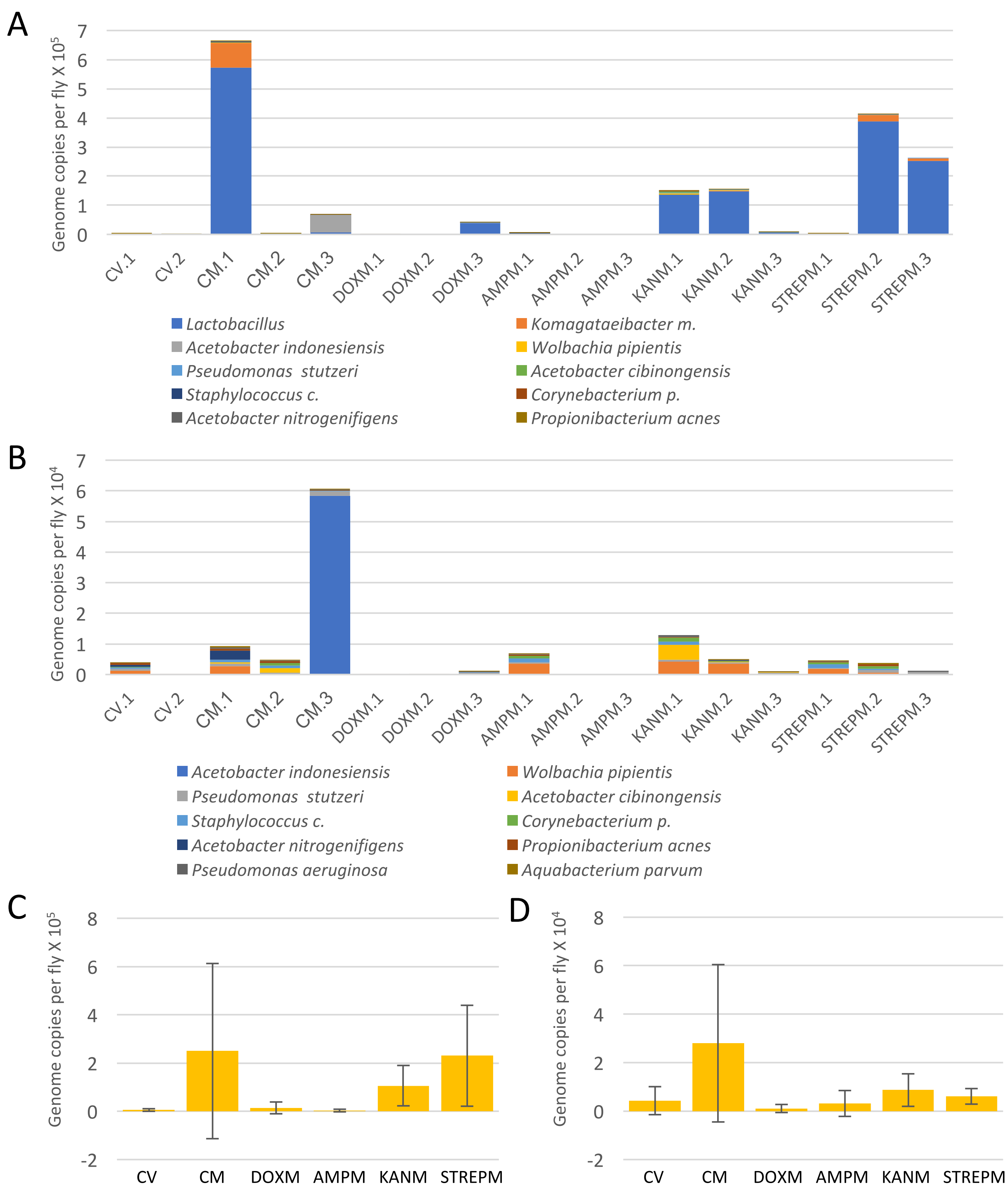

FIGURE S2. Effect of high-concentration antibiotics on mated female life span and identification of outliers. **(A)** Top 10 species including outliers. **(B)** Top 10 species excluding outliers. **(C)** Average total bacteria Including outliers. **(D)** Average total bacteria excluding outliers. CV, control virgins. CM, control mated. DOXM, mated plus doxycycline. AMPM, mated plus ampicillin. KANM, mated plus kanamycin. STREPM, mated plus streptomycin. No statistically significant differences were detected between groups by Kruskal-Wallis test, for species, class or total bacteria.

Table S1. COX proportional hazards analyses

Effect of mifepristone (RU) and sterile condition in mated flies, combining all four experiments:

Call:

coxph(formula = (Surv(Day) ~ RU * Sterile), data = mdata)

n= 790, number of events= 790

	coef	exp(coef)	se(coef)	z	Pr(>|z|)	
RU	-1.3394	0.2620	0.1148	-11.669	< 2x10^{-16}	***
Sterile	0.4249	1.5294	0.1013	4.195	2.73 x10^{-05}	***
RU:Sterile	0.3778	1.4591	0.1473	2.565	0.0103	*

Signif. codes: 0 ‘***’ 0.001 ‘**’ 0.01 ‘*’ 0.05 ‘.’ 0.1 ‘ ’ 1

	exp(coef)	exp(-coef)	lower .95	upper .95
RU	0.262	3.8167	0.2092	0.3281
Sterile	1.529	0.6539	1.2540	1.8652
RU:Sterile	1.459	0.6854	1.0932	1.9473

Effect of mifepristone (RU) and sterile condition in mated flies, comparison of replicates:

Replicate 1 control vs the other groups:

> coxph(formula = (Surv(Day) ~ Cohort), data=mdata)

Call:

coxph(formula = (Surv(Day) ~ Cohort), data = mdata)

	coef	exp(coef)	se(coef)	z	p
Replicate 2 control	0.17753	1.19426	0.09765	1.818	0.0691
Replicate 1 sterile	-0.46390	0.62883	0.10618	-4.369	1.25 x10^{-05}
Replicate 2 sterile	-0.46129	0.63047	0.10372	-4.447	8.70 x10^{-06}

Likelihood ratio test=57.93 on 3 df, p=1.625 x10^{-12}

n= 790, number of events= 790

Replicate 1 sterile vs Replicate 2 sterile:

> coxph(formula = (Surv(Day) ~ Cohort), data=axdata)

Call:

coxph(formula = (Surv(Day) ~ Cohort), data = axdata)

	coef	exp(coef)	se(coef)	z	p
Replicate 2 sterile	-0.002479	0.997524	0.105317	-0.024	0.981

Likelihood ratio test=0 on 1 df, p=0.9812

n= 366, number of events= 366

Effect of *Enterococcus faecalis* and mifepristone, combining all three experiments:

Call:
coxph(formula = (Surv(Day) ~ RU + Dox + Bact + RU:Bact + RU:Dox),
data = alldata)
n= 1608, number of events= 1608

	coef	exp(coef)	se(coef)	z	Pr(>|z|)	
RU	-0.73072	0.48156	0.08861	-8.247	<2 $x10^{-16}$	***
Dox	0.07741	1.08048	0.08636	0.896	0.370	
Bact	0.12750	1.13599	0.08584	1.485	0.137	
RU:Bact	0.14482	1.15584	0.12233	1.184	0.236	
RU:Dox	-0.01379	0.98631	0.12230	-0.113	0.910	

Signif. codes: 0 ‘***’ 0.001 ‘**’ 0.01 ‘*’ 0.05 ‘.’ 0.1 ‘ ’ 1

	exp(coef)	exp(-coef)	lower .95	upper .95
RU	0.4816	2.0766	0.4048	0.5729
Dox	1.0805	0.9255	0.9122	1.2798
Bact	1.1360	0.8803	0.9601	1.3441
RU:Bact	1.1558	0.8652	0.9094	1.4690
RU:Dox	0.9863	1.0139	0.7761	1.2535

Concordance= 0.594 (se = 0.008)
Likelihood ratio test= 192.4 on 5 df, p=<2 $x10^{-16}$
Wald test = 189.7 on 5 df, p=<2 $x10^{-16}$
Score (logrank) test = 197.1 on 5 df, p=<2 $x10^{-16}$

Effect of *Enterococcus faecalis* and mifepristone, comparison of replicates:

Replicate 1 compared to replicates 2 and 3
> coxph(formula = (Surv(Day) ~ Cohort), data=alldata)
Call:
coxph(formula = (Surv(Day) ~ Cohort), data = alldata)

	coef	exp(coef)	se(coef)	z	p
Replicate 2	0.42036	1.52251	0.06243	6.733	1.66 $x10^{-11}$
Replicate 3	0.56607	1.76133	0.06470	8.749	< 2$x10^{-16}$

Likelihood ratio test=85.59 on 2 df, p=< 2.2 $x10^{-16}$
n= 1608, number of events= 1608
